# Homoacetogenic Conversion of Mannitol by the Thermophilic Acetogenic Bacterium *Thermoanaerobacter kivui* Requires External CO_2_

**DOI:** 10.3389/fmicb.2020.571736

**Published:** 2020-09-15

**Authors:** Jimyung Moon, Surbhi Jain, Volker Müller, Mirko Basen

**Affiliations:** Department of Molecular Microbiology and Bioenergetics, Institute of Molecular Biosciences, Johann Wolfgang Goethe University, Frankfurt, Germany

**Keywords:** carbon dioxide reduction, mannitol, acetogenic, thermophilic, *Thermoanaerobacter kivui*, Wood–Ljungdahl pathway

## Abstract

Acetogenic microorganisms utilize organic substrates such as sugars in addition to hydrogen (H_2_) + carbon dioxide (CO_2_). Recently, we reported that the thermophilic acetogenic microorganism *Thermoanaerobacter kivui* is among the few acetogens that utilize the sugar alcohol mannitol, dependent on a gene cluster encoding mannitol uptake, phosphorylation and oxidation of mannitol-1-phosphate to fructose-6-phosphate. Here, we studied mannitol metabolism with resting cells of *T. kivui*; and found that mannitol was “fermented” in a homoacetogenic manner, i.e., acetate was the sole product if HCO_3_^–^ was present. We found an acetate:mannitol ratio higher than 3, indicating the requirement of external CO_2_, and the involvement of the WLP as terminal electron accepting pathway. In the absence of CO_2_ (or bicarbonate, HCO_3_^–^), however, the cells still converted mannitol to acetate, but slowly and with stoichiometric amounts of H_2_ formed in addition, resulting in a “mixed” fermentation. This showed that–in addition to the WLP–the cells used an additional electron sink–protons, making up for the “missing” CO_2_ as electron sink. Growth was 2.5-fold slower in the absence of external CO_2_, while the addition of formate completely restored the growth rate. A model for mannitol metabolism is presented, involving the major three hydrogenases, to explain how [H] make their way from glycolysis into the products acetate or acetate + H_2_.

## Introduction

Acetogens thrive from the formation of acetate from hydrogen (H_2_) + carbon dioxide (CO_2_). Hence, they are an important part of the anaerobic food web, linking primary fermentation to methanogenesis (Schink and Stams, 2006). In addition to H_2_ + CO_2_, most acetogens utilize a variety of “heterotrophic substrates” (Diekert and Wohlfarth, 1994; Schuchmann and Müller, 2016). For example, most acetogens also grow heterotrophically with C6 sugars as substrates, as discovered already in 1942 (Fontaine et al., 1942). Since they convert these to three molecules of acetate as sole major product, acetogens have originally been described as “homoacetogens” (Drake et al., 2008). In “homoacetogenesis,” glucose is oxidized to 2 acetate, 2 CO_2_, yielding 8 reducing equivalents [H] (eq. 1) and 4 ATP (not shown in the equation; for bioenergetics, please see Schuchmann and Müller, 2014).

(1) $${\text{C}}_{6}{\text{H}}_{12}{\text{O}}_{6}+2{\text{H}}_{2}\text{O}\to 2{\text{CH}}_{3}\text{COOH}+2{\text{CO}}_{2}+8\left[\text{H}\right]$$

Importantly and uniquely within the fermentative organisms, homoacetogens then recycle the excess reducing equivalents (“electrons”) in form of 2 NADH and 2 molecules ferredoxin (Fd_red_) by reducing 2 CO_2_ in the Wood–Ljungdahl pathway (WLP) (eq. 2), with *n* ATP being formed in the acetogenic respiratory chain (Schuchmann and Müller, 2014).

(2) $$2{\text{CO}}_{2}+8\text{[H]}\to {\text{CH}}_{3}\text{COOH}+2{\text{H}}_{2}\text{O}$$

In sum, glucose is oxidized to 3 acetates according to eq. 3.

(3) $${\text{C}}_{6}{\text{H}}_{12}{\text{O}}_{6}\to 3{\text{CH}}_{3}\text{COOH}$$

The question now arises how molecules are metabolized that are more reduced, such as the C6 sugar alcohol mannitol. Mannitol, an abundant reserve carbohydrate in brown algae (Adams et al., 2011) has been described as a growth substrate for 8 out of the 47 acetogens that have been sequenced (Moon et al., 2019, and references therein). Mannitol oxidation to acetate yields 10 [H], 2 [H] more than glucose (eq. 4 vs. eq. 1).

(4) $${\text{C}}_{6}{\text{H}}_{14}{\text{O}}_{6}+2{\text{H}}_{2}\text{O}\to 2{\text{CH}}_{3}\text{COOH}+2{\text{CO}}_{2}+10\text{[H]}$$

In mannitol conversion by acetogens, consequently, electrons have to be deposited either internally on an intermediate of the sugar oxidation, yielding a more reduced product than acetate, or on an external electron acceptor. The coupling of mannitol oxidation to the WLP, however, has not been studied in detail in any acetogen.

Here, we describe the catabolism of the thermophilic acetogenic bacterium *Thermoanaerobacter kivui* growing on the sugar alcohol mannitol. We recently characterized the uptake of mannitol by a phosphotransferase system (PTS) and the subsequent conversion of mannitol-1-phosphate by a thermostable mannitol-1-phosphate dehydrogenase in *T. kivui* (Moon et al., 2019). By a variety of physiological experiments with growing cells and cell suspension, we now show unambiguously that *T. kivui* utilizes external CO_2_ as additional electron acceptor during growth on and conversion of mannitol; the biochemical and eco-physiological consequences are discussed.

## Results and Discussion

### Homoacetogenic Conversion of Mannitol Plus CO_2_ in Cell Suspensions

**FIGURE 1:**
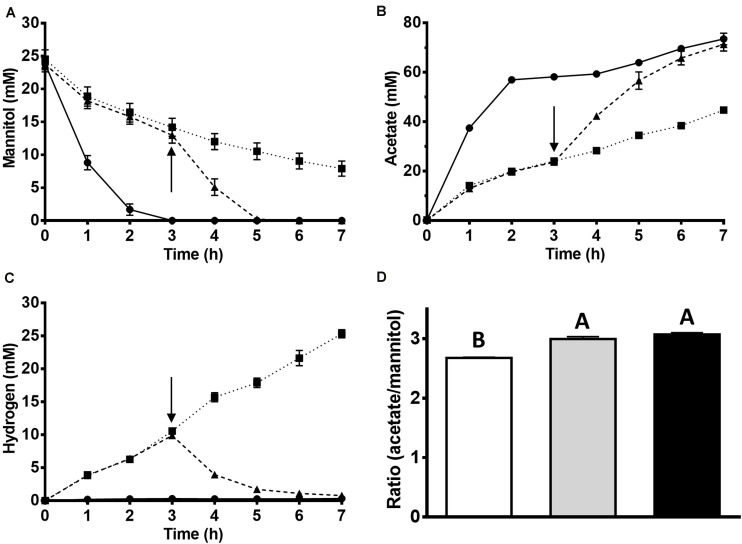
Effect of KHCO_3_ on acetate and hydrogen formation from mannitol by cell suspensions of *T. kivui*. 10 ml of the resting cells (1.0 mg/ml protein) were incubated at 65°C for 7 h under anoxic conditions (N_2_ headspace). 0.8 ml samples were taken for determination of **(A)** mannitol and **(B)** acetate. **(C)** Hydrogen gas was determined by gas chromatography. Cell suspensions were either not supplied with KHCO_3_ (squares), supplied with 54 mM KHCO_3_ after 3 h of incubation (triangles) or supplied with 54 mM KHCO_3_ (circles) from the beginning. The arrow indicates the addition of 54 mM KHCO_3_. **(D)** Ratio of acetate produced to mannitol consumed after 7 h of incubation. White, without KHCO_3_; gray, addition of 54 mM KHCO_3_ at 3 h; black, with 54 mM KHCO_3_ from the beginning. The experiments were performed in biological triplicates. Bars not sharing the same letter indicate a significant difference (*p* ≤ 0.05) according to Tukey’s HSD test.

While homoacetate fermentation theoretically yields three molecules of acetate as sole product from C6 sugars, experimentally, acetate to C6 (fructose or glucose) ratios of 2.6, 2.7, and 2.3–3 have been observed in growing cultures of the acetogens *Moorella thermoacetica* (Fontaine et al., 1942), *Acetobacterium woodii* (Heise et al., 1989) and *T. kivui* (Leigh et al., 1981), respectively. In our hands, non-growing cells of *T. kivui* in concentrated suspensions (which excludes that carbon and reducing equivalents were channeled into biomass), converted glucose to mainly acetate (Supplementary Figure S1), with only minor amounts of H_2_ (0.2 mM; Figure 1C, for comparison calculated as if all H_2_ in was dissolved; *n* H_2_ in headspace/*vol* medium). The resulting acetate:glucose ratio of 2.6 ± 0.1, clearly indicates the involvement of the WLP in the recycling of reduced redox carriers, since the ratio is >2.0. Omitting HCO_3_^–^ (the hydrated, deprotonated form of CO_2_) in the cell suspension experiments did not lead to a significantly different acetate:glucose ratio (Supplementary Figure S1D) and, again, only little H_2_ (1.9 ± 0.5) mM was formed (Supplementary Figure S1C), showing only a minor fraction of the reductant was removed by proton reduction. As expected from thermodynamic considerations, however, the rates of glucose consumption and acetate production decreased by approximately 60%, from −197 ± 14 nmol min^–1^ mg^–1^ (protein) to −71 ± 4 nmol min^–1^ mg^–1^ and 438 ± 47 nmol min^–1^ mg^–1^ (protein) to 199 ± 13 nmol min^–1^ mg^–1^ (Supplementary Figures S1A,B). To directly demonstrate the effect of CO_2_ on glucose conversion, HCO_3_^–^ was added to a subset of cell suspensions after 3 h. The rate of glucose consumption and acetate production increased, and most obviously, intermediately accumulated H_2_ (∼0.5 mM) was re-utilized by the cells.

As mannitol is more reduced than glucose by two electrons, the question arose where the additional electrons go that are transferred to NAD^+^ in the MtlD reaction. One option would be an additional reduced product, such as lactate, H_2_, ethanol or formate. Metabolite analyses in our recent experiments with *T. kivui* growing on mannitol (Moon et al., 2019), however, revealed no major other products. We are aware of only one other study in which products of mannitol utilization in an acetogen, *Sporomusa termitida*, were quantified; and in that organism, acetate was as well the major product, with a slightly lower ratio (2.6 mol per mol mannitol), and with minor amounts of some other products such as propionate or ethanol detected (Breznak et al., 1988). We performed more experiments, actively searching for such reduced compounds using HPLC and GC analyses; however, maximally trace amounts (<0.5 mM lactate or ethanol) were detected in the supernatant of growing or resting cells. Therefore, we hypothesized that CO_2_ present in the medium is the sole major electron acceptor according to eq. 2. Hence, mannitol would be converted to acetate according to eq. 5.

(5) $$4{\text{C}}_{6}{\text{H}}_{14}{\text{O}}_{6}+2{\text{CO}}_{2}\to 13{\text{CH}}_{3}{\text{COO}}^{-}+13{\text{H}}^{+}+2{\text{H}}_{2}\text{O}$$

To prove the involvement of CO_2_, concentrated cell suspensions of *T. kivui* were incubated at 65°C with mannitol in the presence and in the absence of HCO_3_^–^ in the medium. In the control experiment with 54 mM of HCO_3_^–^ present, 23.8 ± 1.5 mM mannitol was rapidly consumed (Figure 1A), and acetate (73.2 ± 4.1 mM) was produced (Figure 1B). No major other product was detected and, consequently, almost all of the reducing equivalents (92 ± 2%) from mannitol oxidation were recovered in the product acetate, even more than in incubations with glucose. Considering mannitol conversion according to eq. 5 and assuming 1/2 molecule of CO_2_ reduced per molecule mannitol, all substrate carbon (mannitol and CO_2_) was re-found in the product acetate (100 ± 2%). The observed acetate:mannitol ratio of 3.1 ± 0.1 (Figure 1D) supports the hypothesis of a homoacetogenic conversion of mannitol, with the need for additional CO_2_, putatively according to eq. 5. This is in contrast to glucose metabolism, where the amount of CO_2_ released from glucose oxidation equals the amount of CO_2_ needed as electron acceptor in the WLP (no net consumption of CO_2_ according to eq. 3).

Therefore, mannitol consumption and conversion to acetate should be more affected than glucose conversion if HCO_3_^–^/CO_2_ is omitted from incubations; and that is what we observed. In the incubations without HCO_3_^–^, less mannitol was consumed (16.6 ± 0.5 mM) and less acetate (44.5 ± 1.4 mM) was produced. The rate of mannitol consumption decreased to a third (from −185 ± 18 nmol min^–1^ mg^–1^ to −58 ± 9 nmol min^–1^ mg^–1^), as the rate of acetate formation did concomitantly (from 472 ± 34 nmol min^–1^ mg^–1^ to 129 ± 11 μmol min^–1^ mg^–1^). Accordingly, the ratio of acetate produced per mannitol in the experiment without HCO_3_^–^ was significantly lower, 2.7 ± 0.0, Figure 1D). Instead, significantly more H_2_ was produced (corresponding to 25.3 ± 1.1 mM if all hydrogen was dissolved, Figure 1C) compared to the corresponding incubations with glucose (1.9 mM ± 1.4 mM). This shows that *T. kivui* used protons as electron acceptors in mannitol metabolism in the absence of external CO_2_/HCO_3_^–^. The metabolism can be seen as a mixed fermentation, with part of the reductant going to protons, similar to what has been observed for sugar oxidation e.g., in *Thermotoga maritima* (Schröder et al., 1994). The other part is still channeled to the WLP, since CO_2_ is released from mannitol oxidation through the PFOR reaction (eq. 6) In conclusion, mannitol metabolism in *T. kivui* cell suspensions in the absence of CO_2_ can be described by eq. 6 (more reductant channeled to protons), eq. 7 (only “extra” reductant from sugar alcohol phosphate oxidation to a sugar phosphate channeled to protons, Supplementary Figure S2), or a mixture thereof.

(6) $${\text{C}}_{6}{\text{H}}_{14}{\text{O}}_{6}+2{\text{H}}_{2}\text{O}\to 2{\text{CH}}_{3}\text{COOH}+2{\text{CO}}_{2}+5{\text{H}}_{2}$$

(7) $${\text{C}}_{6}{\text{H}}_{14}{\text{O}}_{6}\to 3{\text{CH}}_{3}\text{COOH}+{\text{H}}_{2}$$

When HCO_3_^–^ was added to the HCO_3_^–^ free incubations after 3 h, mannitol consumption and acetate production accelerated again (Figures 1A,B). H_2_ that had accumulated intermediately in the absence of HCO_3_^–^ was consumed again after its addition (∼10 mM), leaving only a minor amount (0.8 ± 0.1 mM, Figure 1C). No other major products were observed in any of the incubations, and the reducing equivalents were almost stoichiometrically recovered in the products (92–95% recovery).

### Growth on Mannitol Is CO_2_-Dependent

While the experiments with concentrated cell suspensions directly demonstrated the influence of external HCO_3_^–^/CO_2_ on glucose, but particularly on mannitol conversion (Figure 1 and Supplementary Figure S1), it remained to be tested whether and how this affects growth on both substrates. We hypothesized that growth on both substrates was affected due to thermodynamic reasons, and the effect may be stronger during growth on mannitol. To test this hypothesis, we grew *T. kivui* in defined medium with 25 mM glucose or mannitol under a pure N_2_ atmosphere in the presence or absence of 54 mM KHCO_3_.

**FIGURE 2:**
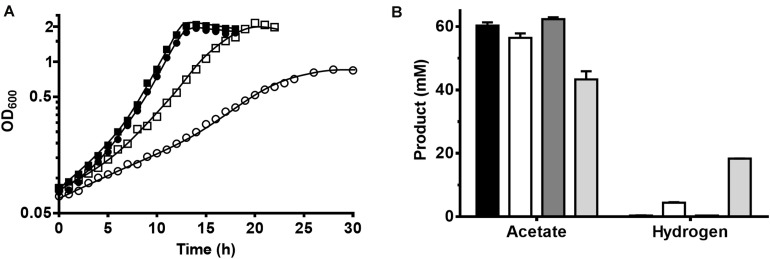
Growth of *T. kivui* on glucose and on mannitol in the presence or absence of carbonate in medium at 65°C. **(A)** Growth of *T. kivui* on 25 mM glucose in carbonate buffered defined medium (black squares), on 25 mM glucose in carbonate free defined medium (white squares), on 25 mM mannitol in carbonate buffered defined medium (black circles), and on 25 mM mannitol in carbonate free defined medium (white circles) at 65°C. The experiments were performed in biological triplicates and one representative growth curve is shown. **(B)** Acetate and hydrogen produced during growth. Black bars, on 25 mM glucose in carbonate buffered defined medium; white bars, on 25 mM glucose in carbonate free defined medium; dark gray bars, on 25 mM mannitol in carbonate buffered defined medium; light gray bars, on 25 mM mannitol in carbonate free defined medium.

Growth on glucose was slowed down in HCO_3_^–^ (and CO_2_) free defined medium, as the doubling time (*t*_*D*_) of *T. kivui* increased from 1.7 ± 0.2 h to 2.9 ± 0.1 h (Figure 2A). An increase in the doubling time (*t*_*D*_) was expected for thermodynamic reasons, the concentration of CO_2_ was much lower–only the CO_2_ released in the PFOR reaction was present. As expected, a more severe effect was observed in the incubations with mannitol, where the *t*_*D*_ increased from 2.0 ± 0.0 to 5.2 ± 0.0 h. The maximum OD_600_ of *T. kivui* cultures grown on mannitol in HCO_3_^–^ -free medium was 0.86 compared to OD_600_ higher than 2.0 in the presence of HCO_3_^–^. Differences were found in the product concentrations as well (Figure 2B). Without HCO_3_^–^, cells grown on glucose produced slightly less acetate (56.4 ± 1.4 mM) than with HCO_3_^–^ (60.3 ± 1.0 mM), and some H_2_ was produced (4.5 ± 0.4 mM). Cells grown on mannitol showed the same tendency, but much bigger differences between incubations were observed with and without HCO_3_^–^. The amount of acetate produced by cells without HCO_3_^–^ reached 43.3 ± 2.6 mM, which is much less compared to those grown in the presence of HCO_3_^–^ (62.3 ± 0.6 mM). Instead, more H_2_ was produced (17.7 ± 1.2 mM vs. 0.4 ± 0.0 mM), as observed in the experiments with the (non-growing) cell suspensions (Figure 1).

One major outcome of the growth experiment was that CO_2_ released from sugar or sugar alcohol oxidation was sufficient to sustain growth, though at significantly decreased growth rates. CO_2_ dependence and fermentation capabilities of acetogens sugar conversion have not been studied much recently. Early evidence for CO_2_-dependence of acetogenic conversion of sugars were obtained in a study from Andreesen et al. (1970) who found that the mesophilic carboxydotroph *Clostridium formicoaceticum* grew only with a long lag phase and to much lower optical densities in the absence of NaHCO_3_. Also, it was shown in the same study that ^14^CO_2_ was incorporated into ^14^C-acetate, with both the methyl and the carbonyl group being labeled, consistent with the utilization of the WLP as terminal electron accepting pathway (Wood et al., 1986). Contrarily, a study from 1996 then revealed that the mesophilic acetogen *Blautia producta* still grew on fructose or xylose in the absence of CO_2_, with molar growth yields reduced by about 30–35%, and [H] channeled into the reduced carbon products succinate and lactate, instead of into H_2_ (Misoph and Drake, 1996). Moreover, the acetate:fructose ratio was below 2, indicating that the WLP was potentially not involved in re-oxidation of reduced electron carriers. Another acetogen, the mesophilic model organism *A. woodii* produces a yet unknown reduced metabolite and less acetate when its Rnf complex is dysfunctional in the absence of Na^+^, or deleted (Heise et al., 1989; Westphal et al., 2018). Acetogens utilize other reduced substrates; alcohols such as methanol or ethanol for example, and the basic metabolic “problem” applies here: Growth on these substrates require additional electron removal. Accordingly, electron removal through the WLP with reduced non-sugar substrates has been proposed e.g., for *A. woodii* growing on methanol (Bache and Pfennig, 1981) ethanol (Buschhorn et al., 1989; Bertsch et al., 2016), or *Acetobacterium carbinolicum* on a variety of alcohols (Eichler and Schink, 1984), with the closed carbon balances indicating CO_2_ utilization in the latter, at least.

So similarly to the cell suspension experiments, *T. kivui* utilized protons as electron acceptors in the absence of CO_2_, supposedly, via the electron-bifurcating hydrogenase, working in confurcating direction. This is slightly different (albeit not contradictory) to our recent observations of a strict dependency of *T. kivui* on the WLP in a strain where the WLP was functionally abolished. The *T. kivui* mutant lacked the hydrogen-dependent CO_2_ reductase (HDCR), the first enzyme of the methyl branch of the WLP (Jain et al., 2020). Cell suspension of that mutant strain also produced H_2_ from glucose in the absence of formate - similar to mannitol conversion in the wild type (Figure 1). Growth, however, was not only significantly impaired as observed here (Figure 2), but completely inhibited, except for when formate was added as additional electron acceptor (Jain et al., 2020). Therefore, we concluded that the WLP as terminal electron accepting pathway is essential for growth of *T. kivui* on all substrates (Jain et al., 2020). Here, we provide evidence that *T. kivui* utilized additional electron acceptors (protons) during growth if forced to do so; but the WLP was still the major electron sink, and [H] removal through proton reduction is not fast enough to keep up the growth rate.

### Formate Stimulates Growth in the Absence of External CO_2_

**FIGURE 3:**
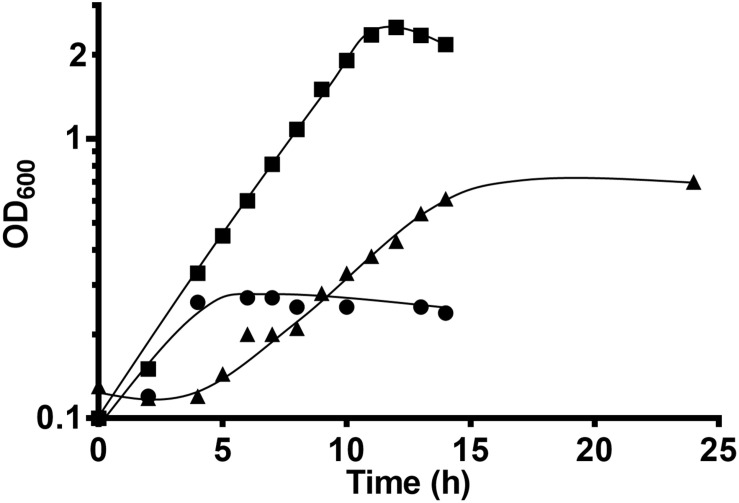
Growth of *T. kivui* on mannitol (25 mM) on defined medium without formate (triangles) or with formate (50 mM, squares) in the absence of HCO_3_^–^/CO_2_, at 65°C. Growth on 50 mM formate (circles) only is shown as a control. Experiments were performed in biological duplicates and a representative growth curve is shown.

Since in the absence of added HCO_3_^–^ (and therefore CO_2_), growth was significantly slowed down, we tested whether external formate could account for the “missing” CO_2_ in wild type *T. kivui*, as recently described for the *T. kivui* HDCR deletion strain (Jain et al., 2020). A growth experiment was set up with *T. kivui* wild type inoculated into CO_2_ and HCO_3_^–^ free defined medium (Figure 3). While in the absence of formate (or CO_2_) again a maximal OD_600_ of only 0.7 was observed and a prolonged doubling time of 5.2 ± 0.2 h, the addition of formate as external electron acceptor increased the maximal OD_600_ to 2.34 and decreased the doubling time to 2.0 ± 0.0 (Figure 3), which corresponds to the growth behavior observed before during growth on mannitol in the presence of CO_2_/HCO_3_^–^ (Moon et al., 2019). Growth on formate as sole substrate contributed only little (Figure 3). 19.7 ± 0.8 mM of mannitol was consumed in the presence of 40.3 ± 2.0 mM formate (which was completely consumed), and 66.0 ± 15.5 mM acetate was produced. We therefore conclude that external formate completely replaced external CO_2_/HCO_3_^–^ during growth on mannitol, constituting the only added electron acceptor. The ability to utilize an electron acceptor other than CO_2_ enhances the metabolic flexibility of acetogens in environments where no or little CO_2_ is present, or to changing environmental conditions. Few additional electron acceptors such as nitrate or aromatic compounds are utilized by some acetogens. In the absence of CO_2_, *A. woodii* for example grows with caffeate as electron acceptor, forming hydrocaffeate as reduced product (Tschech and Pfennig, 1984), potentially giving the organism a metabolic advantage when no CO_2_ is present.

### Mannitol Metabolism in *T. kivui* Is Supported by Its Mode of Energy Conservation

**FIGURE 4:**
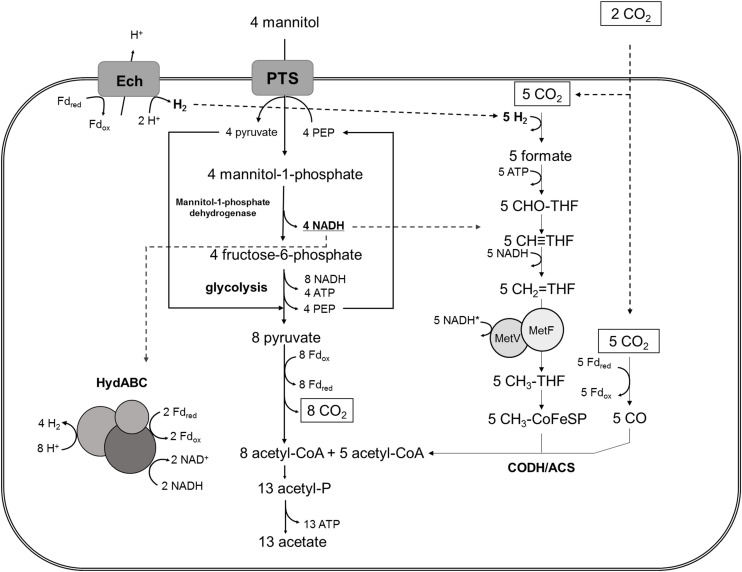
Model for “homoacetogenic” mannitol metabolism in *T. kivui* in the presence of external CO_2_, involving its three major hydrogenases. Reduced ferredoxin (Fd_red_), produced from mannitol oxidation, is oxidized by energy-converting hydrogenase (Ech) and, together with NADH, by electron bifurcating hydrogenase (HydABC) to produce H_2_. The latter is subsequently consumed by hydrogen dependent carbon dioxide reductase (HDCR), containing a hydrogenase subunit, to reduce CO_2_ to formate. In the absence of external CO_2_, a fraction of the reducing equivalents [H] is used to reduce protons to H_2_, likely *via* HydABC. THF, tetrahydrofolate; CODH/ACS, carbon monoxide dehydrogenase/acetyl-CoA synthase; MetV/MetF, methylene-THF reductase (unknown cofactor specificity, *).

In conclusion, the experiments with resting and growing cells of *T. kivui* with and without HCO_3_^–^ showed that the additional electrons from mannitol oxidation were channeled into the WLP for CO_2_ fixation. In the absence of CO_2_ in the medium, additionally protons were reduced to H_2_ (approximately according to eq. 8), but growth and mannitol conversion were significantly reduced. Based on these observations and on the genome model, the following model for mannitol metabolism in *T. kivui* in the presence of external CO_2_ is postulated (Figure 4). Four (molecules of) mannitol are taken up and phosphorylated by a PTS system. Then, four mannitol-1-phosphate are oxidized to four fructose-6-phosphate, yielding 4 NADH. Glycolysis and PFOR yield 8 acetyl-coenzyme A, which is further converted to acetate, 8 CO_2_, 8 NADH and 8 Fd_red_. In the presence of external CO_2_, the reductant (in form of 8 NADH and 8 Fd_red_) is utilized to reduce CO_2_ to acetate. We assume the WLP needs 1 H_2_ for the HDCR, two NADH and 1 Fd_red_ (Hess et al., 2014; Basen and Müller, 2017). When it is run four times to reduce the 8 CO_2_ produced by PFOR, and then another time to reduce 2 additional CO_2_, the redox carriers are not balanced, with 2 spare NADH and 3 spare Fd_red_ on the one hand, and 5 H_2_ needed on the other hand. Redox balancing could be explained by the involvement of energy-converting hydrogenases (Ech), producing 1 H_2_ from 1 Fd_red_, and the electron-bifurcating hydrogenase, producing 4 H_2_ from the remaining 2 NADH and 2 Fd_red_ (Figure 4; Hess et al., 2014; Basen and Müller, 2017).

Accordingly, the involvement of two hydrogenases in redox carrier oxidation may also explain the production of H_2_ in the absence of CO_2_ by *T. kivui* cells, the electron-bifurcating hydrogenase (HydABC) and the membrane-bound Ech, oxidizing the accrued reduced electron carriers, NADH and Fd_red_ or only Fd_red_, respectively. Fd_red_ may also serve as physiological electron donor for HDCR (containing the third hydrogenase involved) as in *A. woodii* (Schuchmann and Müller, 2013). H_2_ production from sugar involving an electron-confurcating hydrogenase is likely widespread among fermentative anaerobes (Schut and Adams, 2009; Verbeke et al., 2013; Zheng et al., 2014; Cha et al., 2016). The essential principle here is the involvement an electron-bifurcating hydrogenase operating reverse (confurcating) direction, and concomitantly oxidizing NADH and Fd_red_, as originally described in the thermophilic fermentative bacterium *T. maritima* (Schut and Adams, 2009). In acetogens, the intermediate accumulation of only small concentrations of H_2_ during sugar metabolism has been demonstrated, for the thermophile *M. thermoacetica* (Kellum and Drake, 1984), and more recently in the mesophile *A. woodii* (Wiechmann et al., 2020). In the latter, the electron-bifurcating hydrogenase has been shown to be involved in a variation of H_2_ cycling (see below), which had also been proposed for *M. thermoacetica* (Wang et al., 2013). Our genetic experiments with *T. kivui* (Jain et al., 2020) as well as the experiments presented with excess [H] from mannitol presented herein now go in line with the earlier observations with *M. thermoacetica*, suggesting that both organisms may have a similar metabolism during growth on sugars or sugar alcohols. The observed stoichiometric coupling of mannitol oxidation to CO_2_ reduction the WLP, with the (proposed) involvement of Ech during heterotrophic growth, indicate that the module of reductant removal (the WLP) and the mode of energy conservation (chemiosmosis *via* Ech) may have been maintained in (the thermophilic) acetogens as conservative traits, enabling the ability to adapt to different electron donors. Indeed both, membrane-bound hydrogenases (Schut et al., 2016) and the WLP (Weiss et al., 2016) have been considered ancient metabolic modules. The interplay of the two hydrogenases (Ech and electron-bifurcating hydrogenase) may enable *T. kivui* the adaptation to substrates at different redox states, since different ratios of NADH and Fd_red_ may be achieved, and this remains subject of future studies.

## Materials and Methods

### Growth Experiments

The wild type *T. kivui* strain LKT-1 (DSM2030) was cultivated under strict anoxic conditions at 65°C in either complex or carbonate buffered defined medium as described previously (Moon et al., 2019). Carbonate free medium was prepared as carbonate buffered defined medium, but no KHCO_3_ was added and the medium was flushed with 100% N_2_. To account for traces of CO_2_ in the carbonate free medium, the growth experiments toward the effect of formate (Figure 3) were carried out with medium that has been boiled (autoclaved) to remove traces of CO_2_, and then flushed with N_2_ (CO_2_-free medium). For determining the growth behavior, cultures were inoculated to an optical density of ∼0.1 from a pre-culture grown on the same substrate (glucose or mannitol), and then incubated at 65°C under slow shaking. Growth was monitored by measuring the optical density of subsamples at 600 nm in cuvettes with 1 cm light path.

### Experiments With Resting Cells

A 500 ml cultures of *T. kivui* were grown in complex or defined medium to late exponential growth phase (OD_600_ of 1.7 to 2.3) and then harvested by centrifugation (Avanti^TM^J-25 and JA-10 Fixed-Angle Rotor; Beckman Coulter, Brea, CA, United States) at 7,000 × *g* and 4°C for 10 min. The harvested cells were washed with 30 ml of the respective medium by centrifugation at 8,500 rpm (5948 × *g*) and 4°C for 10 min (Avanti^TM^J-25 and JA-25.50 Fixed-Angle Rotor; Beckman Coulter, Brea, CA, United States). Then, the cells were resuspended in 5 ml of the respective medium and kept in 16 ml Hungate tubes. Resuspended cells were distributed into in Hungate tubes to a final volume of 10 mL and a final protein concentration of 10 mg ml^–1^. All the steps were performed under strictly oxygen free conditions in an anoxic chamber (Coy Laboratory Products, Grass Lake, MI, United States) filled with N_2_/CO_2_ (80/20; v/v) for carbonate medium or with 100% N_2_ for carbonate free medium. As substrate, 25 mM glucose or 25 mM mannitol was added to the resting cells. The experiment started with incubation at 65°C in water bath with shaking (150 rpm). 0.8 ml subsamples were taken for determination of protein, substrate and product concentration. The total protein concentration in the cell suspension was measured using the method by Schmidt et al. (1963).

### Analysis of Substrate Decrease and Product Formation

H_2_, alcohol and organic acid concentrations were determined by gas chromatography as described previously (Weghoff and Müller, 2016). The concentrations of glucose and mannitol were determined by high performance liquid as described previously (Moon et al., 2019). Lactic acid and formic acid concentrations were determined using test kits (R-Biopharm AG, Darmstadt, Germany) according to supplier’s instruction.

### Statistical Analysis

The ratio of acetate/substrate of *T. kivui* in cell suspension experiments was evaluated by comparing the average values of three biological replicates. For comparison of multiple groups, one-way analysis of variance (ANOVA) with Tukey’s HSD test was carried out by the XLStat software (Version 2019, Addinsoft, New York, NY, United States).

## Data Availability Statement

All datasets presented in this study are included in the article/Supplementary Material.

## Author Contributions

VM and MB designed the study. JM and SJ performed the experiments and prepared the figures. JM, VM, and MB wrote the manuscript. All authors analyzed the data.

## Conflict of Interest

The authors declare that the research was conducted in the absence of any commercial or financial relationships that could be construed as a potential conflict of interest.

## References

[B1] Adams J. M. Ross A. B. Anastasakis K. Hodgson E. M. Gallagher J. A. Jones J. M. (2011). Seasonal variation in the chemical composition of the bioenergy feedstock *Laminaria digitata* for thermochemical conversion. *Bioresour. Technol.* 102 226–234. 10.1016/j.biortech.2010.06.152 20685112

[B2] Andreesen J. R. Gottschalk G. Schlegel H. G. (1970). Clostridium formicoaceticum nov. *spec. Isolation, description and distinction from C. aceticum and C. thermoaceticum*. *Arch. Mikrobiol.* 72 154–174. 10.1007/bf00409521 4918913

[B3] Bache R. Pfennig N. (1981). Selective isolation of *Acetobacterium woodii* on methoxylated aromatic acids and determination of growth yields. *Arch. Microbiol.* 130 255–261. 10.1007/bf00459530

[B4] Basen M. Müller V. (2017). “Hot” acetogenesis. *Extremophiles* 21 15–26. 10.1007/s00792-016-0873-3 27623994

[B5] Bertsch J. Siemund A. L. Kremp F. Müller V. (2016). A novel route for ethanol oxidation in the acetogenic bacterium *Acetobacterium woodii*: the acetaldehyde/ethanol dehydrogenase pathway. *Environ. Microbiol.* 18 2913–2922. 10.1111/1462-2920.13082 26472176

[B6] Breznak J. A. Switzer J. M. Seitz H. J. (1988). *Sporomusa termitida* sp. *nov., an H*2/CO2-utilizing acetogen isolated from termites. *Arch. Microbiol.* 150 282–288. 10.1007/bf00407793

[B7] Buschhorn H. Dürre P. Gottschalk G. (1989). Production and utilization of ethanol by the homoacetogen *Acetobacterium woodii*. *Appl. Environ. Microbiol.* 55 1835–1840. 10.1128/aem.55.7.1835-1840.1989 16347978 PMC202959

[B8] Cha M. Chung D. Westpheling J. (2016). Deletion of a gene cluster for [Ni-Fe] hydrogenase maturation in the anaerobic hyperthermophilic bacterium *Caldicellulosiruptor bescii* identifies its role in hydrogen metabolism. *Appl. Microbiol. Biotechnol.* 100 1823–1831. 10.1007/s00253-015-7025-z 26536872

[B9] Diekert G. Wohlfarth G. (1994). Metabolism of homoacetogens. *Antonie Van Leeuwenhoek* 66 209–221. 10.1007/bf00871640 7747932

[B10] Drake H. L. Gössner A. S. Daniel S. L. (2008). Old acetogens, new light. *Ann. N.Y. Acad. Sci.* 1125 100–128. 10.1196/annals.1419.016 18378590

[B11] Eichler B. Schink B. (1984). Oxidation of primary aliphatic alcohols by *Acetobacterium carbinolicum* sp. nov., a homoacetogenic anaerobe. *Arch. Microbiol.* 140 147–152. 10.1007/bf00454917

[B12] Fontaine F. E. Peterson W. H. Mccoy E. Johnson M. J. Ritter G. J. (1942). A new type of glucose fermentation by *Clostridium thermoaceticum*. *J. Bacteriol.* 43 701–715. 10.1128/jb.43.6.701-715.1942 16560531 PMC373636

[B13] Heise R. Müller V. Gottschalk G. (1989). Sodium dependence of acetate formation by the acetogenic bacterium *Acetobacterium woodii*. *J. Bacteriol.* 171 5473–5478. 10.1128/jb.171.10.5473-5478.1989 2507527 PMC210386

[B14] Hess V. Poehlein A. Weghoff M. C. Daniel R. Müller V. (2014). A genome-guided analysis of energy conservation in the thermophilic, cytochrome-free acetogenic bacterium *Thermoanaerobacter kivui*. *BMC Genomics* 15:1139. 10.1186/1471-2164-15-1139 25523312 PMC4320612

[B15] Jain S. Dietrich H. M. Müller V. Basen M. (2020). Formate Is required for growth of the thermophilic acetogenic bacterium *Thermoanaerobacter kivui* lacking hydrogen-dependent carbon dioxide reductase (HDCR). *Front. Microbiol.* 11:59. 10.3389/fmicb.2020.00059 32082286 PMC7005907

[B16] Kellum R. Drake H. L. (1984). Effects of cultivation gas phase on hydrogenase of the acetogen *Clostridium thermoaceticum*. *J. Bacteriol.* 160 466–469. 10.1128/jb.160.1.466-469.1984 6434525 PMC214747

[B17] Leigh J. A. Mayer F. Wolfe R. S. (1981). *Acetogenium kivui*, a new thermophilic hydrogen-oxidizing, acetogenic bacterium. *Arch. Microbiol.* 129 275–280. 10.1007/bf00414697

[B18] Misoph M. Drake H. L. (1996). Effect of CO2 on the fermentation capacities of the acetogen *Peptostreptococcus productus* U-1. *J. Bacteriol.* 178:3140. 10.1128/jb.178.11.3140-3145.1996 8655492 PMC178064

[B19] Moon J. Henke L. Merz N. Basen M. (2019). A thermostable mannitol-1-phosphate dehydrogenase is required in mannitol metabolism of the thermophilic acetogenic bacterium *Thermoanaerobacter kivui*. *Environ. Microbiol.* 21 3728–3736. 10.1111/1462-2920.14720 31219674

[B20] Schink B. Stams A. (2006). “Syntrophism among prokaryotes,” in *The Prokaryotes - A handbook on the biology of bacteria*, 3rd Edn, eds Dworkin M. Falkow S. Rosenberg E. Schleifer K. H. Stackebrandt E. (Berlin: Springer Science+Business Media, LLC), 309–336. 10.1007/0-387-30742-7_11

[B21] Schmidt K. Jensen S. L. Schlegel H. (1963). Die Carotinoide der *Thiorhodaceae*. *Arch. Mikrobiol.* 46 117–126. 10.1007/bf00408204 14044829

[B22] Schröder C. Selig M. Schönheit P. (1994). Glucose fermentation to acetate, CO2 and H2 in the anaerobic hyperthermophilic eubacterium *Thermotoga maritima*: involvement of the Embden-Meyerhof pathway. *Arch. Microbiol.* 161 460–470. 10.1007/bf00307766

[B23] Schuchmann K. Müller V. (2013). Direct and reversible hydrogenation of CO2 to formate by a bacterial carbon dioxide reductase. *Science* 342 1382–1385. 10.1126/science.1244758 24337298

[B24] Schuchmann K. Müller V. (2014). Autotrophy at the thermodynamic limit of life: a model for energy conservation in acetogenic bacteria. *Nat. Rev. Microbiol.* 12 809–821. 10.1038/nrmicro3365 25383604

[B25] Schuchmann K. Müller V. (2016). Energetics and application of heterotrophy in acetogenic bacteria. *Appl. Environ. Microbiol.* 82 4056–4069. 10.1128/aem.00882-16 27208103 PMC4959221

[B26] Schut G. J. Adams M. W. W. (2009). The iron-hydrogenase of *Thermotoga maritima* utilizes ferredoxin and NADH synergistically: a new perspective on anaerobic hydrogen production. *J. Bacteriol.* 191 4451–4457. 10.1128/jb.01582-08 19411328 PMC2698477

[B27] Schut G. J. Zadvornyy O. Wu C.-H. Peters J. W. Boyd E. S. Adams M. W. W. (2016). The role of geochemistry and energetics in the evolution of modern respiratory complexes from a proton-reducing ancestor. *Biochim.Biophys. Acta Bioenerg.* 1857 958–970. 10.1016/j.bbabio.2016.01.010 26808919

[B28] Tschech A. Pfennig N. (1984). Growth yield increase linked to caffeate reduction in *Acetobacterium woodii*. *Arch. Microbiol.* 137 163–167. 10.1007/bf00414460

[B29] Verbeke T. J. Zhang X. Henrissat B. Spicer V. Rydzak T. Krokhin O. V. (2013). Genomic evaluation of *Thermoanaerobacter* spp. for the construction of designer co-cultures to improve lignocellulosic biofuel production. *PLoS One* 8:e59362. 10.1371/journal.pone.0059362 23555660 PMC3608648

[B30] Wang S. N. Huang H. Y. Kahnt J. Thauer R. K. (2013). A reversible electron-bifurcating ferredoxin- and NAD-dependent [FeFe]-hydrogenase (HydABC) in *Moorella thermoacetica*. *J. Bacteriol.* 195 1267–1275. 10.1128/jb.02158-12 23316038 PMC3591994

[B31] Weghoff M. C. Müller V. (2016). CO metabolism in the thermophilic acetogen *Thermoanaerobacter kivui*. *Appl. Environ. Microbiol.* 82 2312–2319. 10.1128/aem.00122-16 26850300 PMC4959504

[B32] Weiss M. C. Sousa F. L. Mrnjavac N. Neukirchen S. Roettger M. Nelson-Sathi S. (2016). The physiology and habitat of the last universal common ancestor. *Nat. Microbiol.* 1 16116. 10.1038/nmicrobiol.2016.116 27562259

[B33] Westphal L. Wiechmann A. Baker J. Minton N. P. Müller V. (2018). The Rnf complex Is an energy-coupled transhydrogenase essential to reversibly Link cellular NADH and ferredoxin pools in the acetogen *Acetobacterium woodii*. *J. Bacteriol.* 200:e00357-18. 10.1128/JB.00357-18 PMC6182241 30126940

[B34] Wiechmann A. Ciurus S. Oswald F. Seiler V. N. Müller V. (2020). It does not always take two to tango: “Syntrophy” via hydrogen cycling in one bacterial cell. *ISME J.* 14 1561–1570. 10.1038/s41396-020-0627-1 32203116 PMC7242416

[B35] Wood H. G. Ragsdale S. W. Pezacka E. (1986). The acetyl-CoA pathway of autotrophic growth. *FEMS Microbiol. Lett.* 39 345–362. 10.1111/j.1574-6968.1986.tb01865.x

[B36] Zheng Y. N. Kahnt J. Kwon I. H. Mackie R. I. Thauer R. K. (2014). Hydrogen formation and its regulation in *Ruminococcus albus*: Involvement of an electron-bifurcating [FeFe]-hydrogenase, of a non-electron-bifurcating [FeFe]-hydrogenase, and of a putative hydrogen-sensing [FeFe]-hydrogenase. *J. Bacteriol.* 196 3840–3852. 10.1128/jb.02070-14 25157086 PMC4248817

