# Supplementary material for: Homoacetogenic Conversion of Mannitol by the Thermophilic Acetogenic Bacterium Thermoanaerobacter kivui Requires External CO2
Source: Front Microbiol. 2020 Sep 15;11:571736. doi: 10.3389/fmicb.2020.571736 (PMC7522397; doi:10.3389/fmicb.2020.571736)
Supplement: Supplementary file 1 [file Data_Sheet_1.pdf]

Supplementary Material

for the manuscript

Homoacetogenic conversion of mannitol by the thermophilic acetogenic bacterium *Thermoanaerobacter kivui* requires external CO_2

Jimyung Moon[1], Surbhi Jain[1], Volker Müller[1] and Mirko Basen[1,2]*

[1]Department of Molecular Microbiology & Bioenergetics, Institute of Molecular Biosciences, Johann Wolfgang Goethe University, Max-von-Laue Str. 9, D-60438 Frankfurt/Main, Germany

[2]Current address: University of Rostock, Institute of Biological Sciences, Albert-Einstein Str. 3, 18051 Rostock, Germany

*** Correspondence:**

Mirko Basen
mirko.basen@uni-rostock.de

Keywords: carbon dioxide reduction, mannitol, acetogenic, thermophilic, *Thermoanaerobacter kivui*, Wood-Ljungdahl pathway

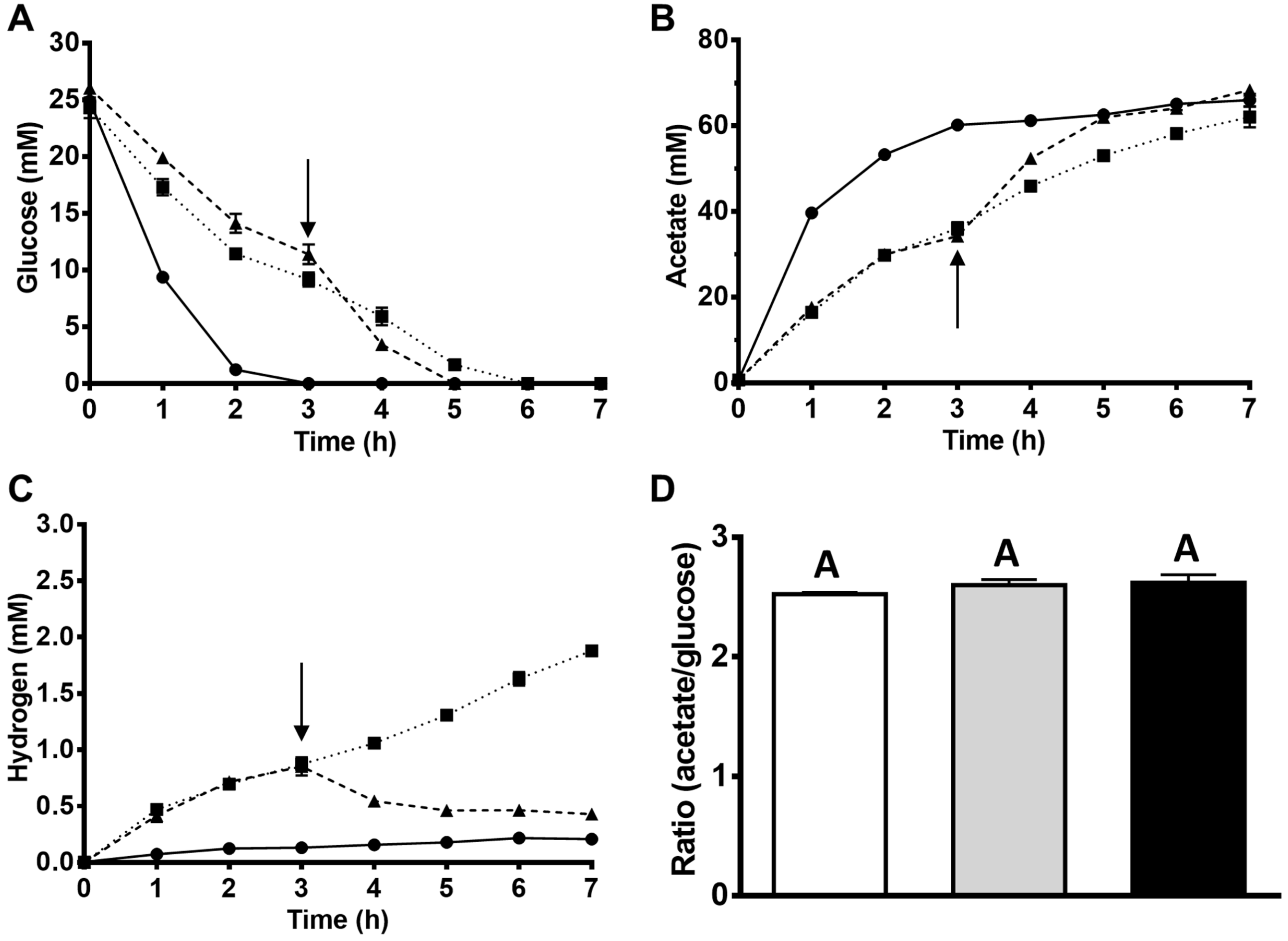

SUPPLEMENTARY FIGURE 1 | Effect of $KHCO_3$ on acetate and hydrogen formation from glucose by *T. kivui*. 10 ml cell suspensions (1.0 mg/ml protein) were incubated at 65°C for 7 hours under anoxic conditions (N_2 headspace). 0.8 ml samples were taken for determination of (A) glucose and (B) acetate. (C) Hydrogen gas was determined by gas chromatography. Cell suspensions were either not supplied with $KHCO_3$ (squares), supplied with 54 mM $KHCO_3$ after 3 h of incubation (triangles) or supplied with 54 mM $KHCO_3$ from the beginning (circles). The arrow indicates the addition of 54 mM $KHCO_3$. (D) Ratio of acetate produced to mannitol consumed after 7 hours of incubation. White, without $KHCO_3$; grey, addition of 54 mM $KHCO_3$ after 3 h of incubation; black, with 54 mM $KHCO_3$ from start. The experiments were performed in biological triplicates. Bars sharing the same letter are not significantly different ($p > 0.05$) according to Tukey's HSD test.

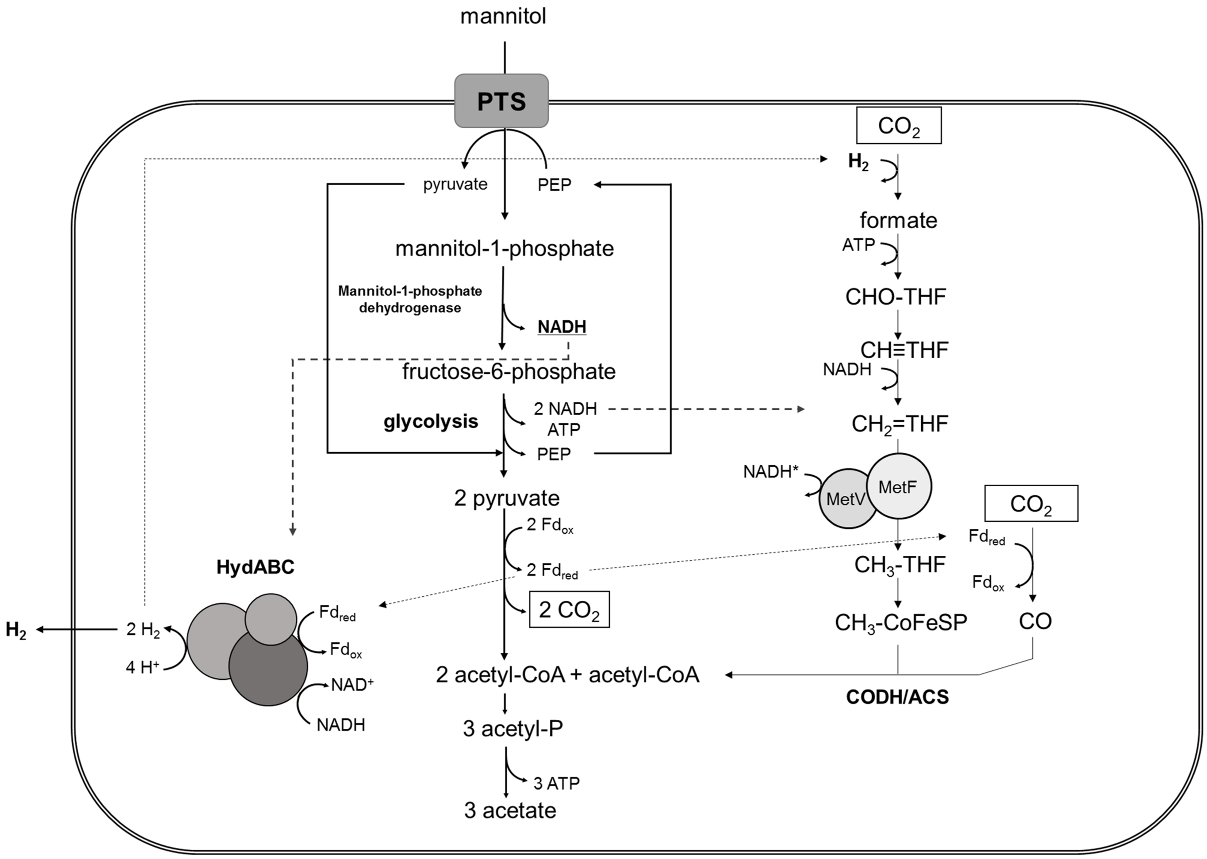

SUPPLEMENTARY FIGURE 2 | Model for mannitol metabolism in *T. kivui* in the absence of external CO_2, according to eq. 7 (most reductant channelled through WLP). Note the differences to Fig. 5. Electron bifurcating hydrogenase (HydABC) may be the only hydrogenase to produce H_2 in the absence of external CO_2, energy converting hydrogenase (Ech) may be dispensable. Part of the H_2 is subsequently consumed by hydrogen dependent carbon dioxide reductase (HDCR), part is released, leaving protons as terminal electron acceptors for mannitol oxidation in *T. kivui*. THF, tetrahydrofolate; CODH/ACS, carbon monoxide dehydrogenase / acetyl-CoA synthase; MetV/MetF, methylene-THF reductase (unknown cofactor specificity, *). Reduced ferredoxin (Fd_{red}),
